# Multiscale mechanobiology: mechanics at the molecular, cellular, and tissue levels

**DOI:** 10.1186/2045-3701-3-25

**Published:** 2013-06-03

**Authors:** Chin-Lin Guo, Nolan C Harris, Sithara S Wijeratne, Eric W Frey, Ching-Hwa Kiang

**Affiliations:** 1 Department of Bioengineering and Department of Applied Physics, California Institute of Technology, MC 138–78, Pasadena, CA 91125, USA; 2 Department of Physics and Astronomy, Rice University, Houston, TX, USA; 3 Department of Bioengineering, Rice University, Houston, TX, USA

**Keywords:** Mechanics, Mechanical force, Biomolecules, Proteins, DNA, Cells, Tissues, Single-molecule manipulation, Atomic force microscopy, Micro-patterning

## Abstract

Mechanical force is present in all aspects of living systems. It affects the conformation of molecules, the shape of cells, and the morphology of tissues. All of these are crucial in architecture-dependent biological functions. Nanoscience of advanced materials has provided knowledge and techniques that can be used to understand how mechanical force is involved in biological systems, as well as to open new avenues to tailor-made bio-mimetic materials with desirable properties.

In this article, we describe models and show examples of how force is involved in molecular functioning, cell shape patterning, and tissue morphology.

## Review

### Introduction

Life relies on the ability of size and shape control at different scales. At the molecular and sub-cellular levels, chemical signaling relies on conformational changes of molecules. Mutations leading to abnormal conformational changes often cause diseases such as tumors and tissue malfunction. At the cellular and tissue levels, the ability of cells to form specific shapes is of vital importance [1,2]. This ability appears to control the fate of cells and tissues. For example, it has been shown that controlling cell shape on micro-fabricated devices can induce cell apoptosis [3], direct cell migration [4], and stem cells differentiation [5]. All of these functions are important in normal tissue development and homeostasis.

From a theoretical point of view, the control of molecular conformation, cell shape, and tissue morphology relies on how mechanical forces are created, distributed, and transmitted. There is a growing interest in the role of mechanical force in tissue development, remodeling, regeneration, and tumorigenesis [6-10]. In most cases, force is transmitted through filaments such as actomyosin bundles inside the cells and collagen fibers outside the cells. The spatial scales of these filaments can be as small as nanometers, while their integrated, mechanical influence on biological systems can be as large as the size of an organ. To understand how the mechanical properties of filamentous molecules at nanometer scales affect the structure and function of biological systems, lessons learned from nanoscience can be applied. In addition, recent advances in nanomaterial sciences open a new door for customized bio-mimetic materials with desired structures and mechanical properties [11].

Understanding how cells create, distribute, transmit, and use forces is essential for using nanomaterials technology in biological systems. At the cellular level, the creation of force within single cells depends on the orientation and distribution of cytoskeleton proteins, such as actomyosin filaments, the organization of which is further regulated by chemical signaling that relies on conformational changes of molecules. Likewise, the propagation of force within tissues is parameterized by the distribution and the orientation of extracellular matrix (ECM) molecules.

### Molecular mechanics

#### Polymer physics models of biomolecules

**Figure 1:**
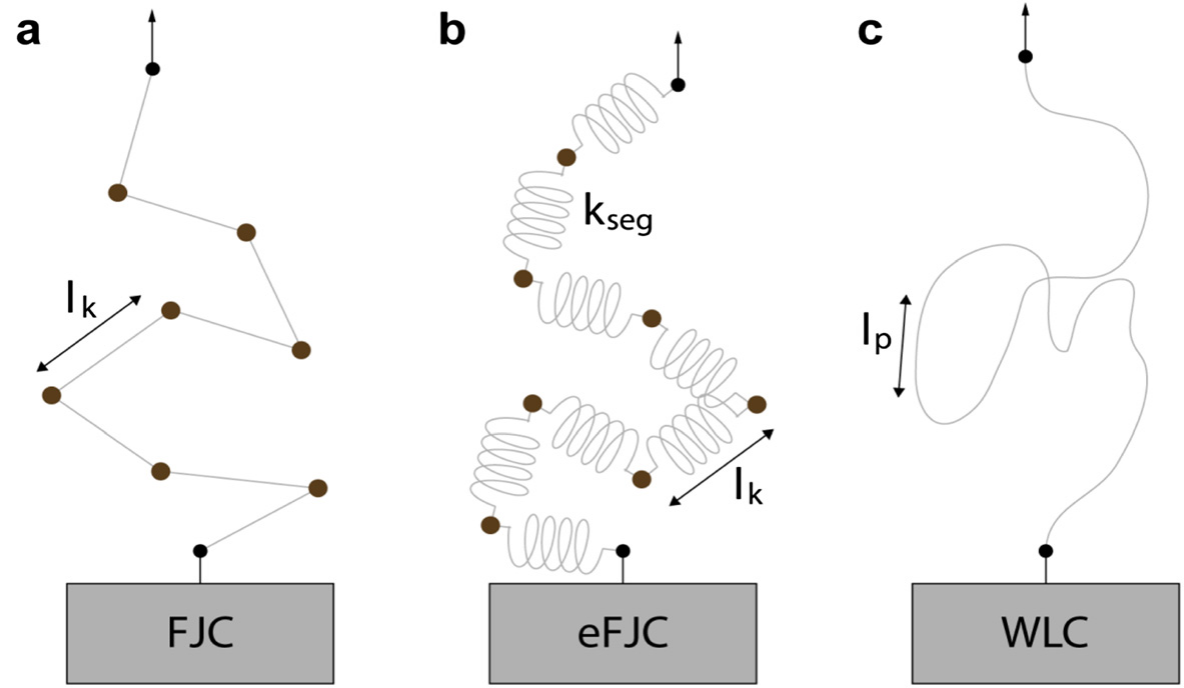
**Polymer elasticity models commonly used in single molecule manipulation. a**, The FJC model consists of segments of length *l*_*k*_ connected via freely-rotating joints. **b**, The eFJC model consists of elastic segments with segment elasticity *k*_*seg*_. **c**, The WLC model describes a polymer molecule as a flexible rod with stiffness defined by the persistence length *l*_*p*_.

The mechanical properties of proteins and DNA can be described using polymer physics models such as the freely-jointed chain (FJC) model and the wormlike chain (WLC) model. The FJC model assumes a polymer chain consisting of *n* segments of characteristic length *l*_*k*_ (Kuhn length) connected via freely-rotating joints (Figure 1a) [12]. The FJC model accounts for the entropic elasticity of the polymer chain up to the contour length, *l*_*c*_=*nl*_*k*_. At high forces, a molecule may be stretched beyond its contour length. Such overstretching transitions can be described by extensible FJC (eFJC) [13,14], which takes into account the additional extension by modeling each segment as an elastic spring with segment elasticity *k*_*seg*_ (Figure 1b). The WLC model treats a polymer molecule as a homogenous elastic rod, or a wormlike chain, characterized by its contour length, *l*_*c*_, and persistence length, *l*_*p*_ (Figure 1c) [12]. The persistence length *l*_*p*_ defines the bending stiffness of the polymer. In single molecule experiments, the force-extension curves are fitted with these polymer physics models to determine the elasticity of the molecules. Typically, single-stranded DNA (ssDNA) is best described by the FJC model, whereas double-stranded DNA (dsDNA) and proteins can be characterized by the WLC model.

#### Stretching reveals folding-refolding characteristics of titin I27 domain

**Figure 2:**
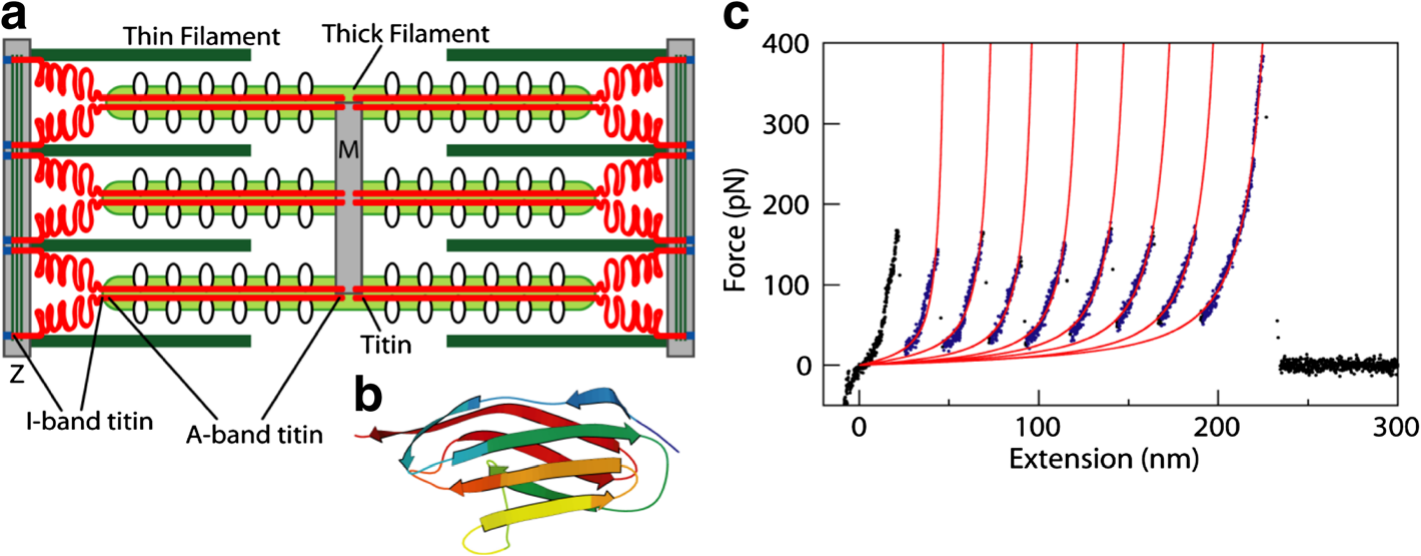
**Human cardiac protein titin. a**, A schematic of titin in the sarcomere. **b**, Structure of I27 domain of titin (Protein Data Bank, ID 1TIT). **c**, Typical force-extension data of titin I27. Curves are WLC model fits to each individual domain stretching event. The force-extension regions over which the WLC model was fit are in blue.

To illustrate how force measurements of molecules are done to obtain the parameters that define the mechanical properties of molecules, we use single molecule force studies of a protein with repeated units. The single molecule manipulation studies are performed using an engineered polyprotein consisting of repeats of the I27 domain of human cardiac titin. Titin is a giant muscle protein of 1 μm in length and 3 MDa in size, found in the striated cardiac and skeletal muscle tissue [15]. Titin molecules span half the sarcomere, from the Z-disc to the M-line, constituting a third of the sarcomeric filament system that binds to both the thick and thin filaments [16]. Figure 2a shows a schematic of the cardiac sarcomere. Titin is divided into an extensible I-band region, which is responsible for the protein's elasticity, and an inextensible A-band region, which functions as a stiff scaffold. Titin has a modular architecture in which both regions are composed primarily of repeats of Immunoglobulin (Ig)-like and Fibronectin type 3 (FN3)-like domains [17]. These domains exhibit a *β*-barrel structure formed from seven anti-parallel *β*-strands. Domains are linked to neighboring domains via an elastic linker region, which is thought to be the main source contributing to the overall flexibility of the chain [18]. The A-band and the I-band are composed primarily of FN3 domains and the stronger Ig domains, respectively. Mechanical stability dictates the arrangement of domains in the I-band, with the weakest Ig domains near the Z-line (proximal Ig region) and the most mechanically stable domains near the M-line (distal Ig region) [19].

The mechanical nature of titin's function makes it particularly suitable for single molecule stretch-relaxation studies [20-26]. In particular, the I27 domain of titin, which was the first structurally determined Ig domain from titin's I-band (Figure 2b) [16], has been widely studied using single molecule manipulation experiments. In these experiments, a biomolecule attaches to the AFM tip and substrate and is stretched as the piezoelectric transducer moves the substrate surface away from the tip, thereby increasing the molecular end-to-end distance. This stretching results in a negative cantilever deflection, followed by an abrupt jump back to the cantilever equilibrium position when one of the domain unfolds or the molecule detaches from the tip [27]. Figure 2c shows a force-extension curve from an AFM stretching experiment, in which each I27 force peak, representing an individual domain stretching event, was fit with the WLC model (red lines). Using nonequilibrium single molecule measurements and Jarzynski’s equality, the free energy surface of both mechanical stretching and unfolding of the I27 domain of human cardiac titin can be reconstructed [22]. Quantitative information about the free energy of unfolding of I27 may allow us to quantify the protein folding free energy landscape, and therefore, to predict the pathways of biological interactions.

#### Mechanical melting of DNA exhibits unique overstretching transitions

There has been renewed interest in understanding the details of thermodynamics and kinetics of DNA melting due to recent advances in both single molecule experimental techniques [13,28-34] and theoretical modeling methods [35-37]. DNA's mechanical properties influence a variety of its biological functions such as how it wraps around histones, packs into phage heads, and interacts with proteins [13]. It is believed that many biological machines depend on the mechanical properties of double-stranded DNA (dsDNA) [30,35]. These mechanical properties can be exploited by novel therapeutics whose design is guided by information extractable from single molecule force measurements [38,39].

**Figure 3:**
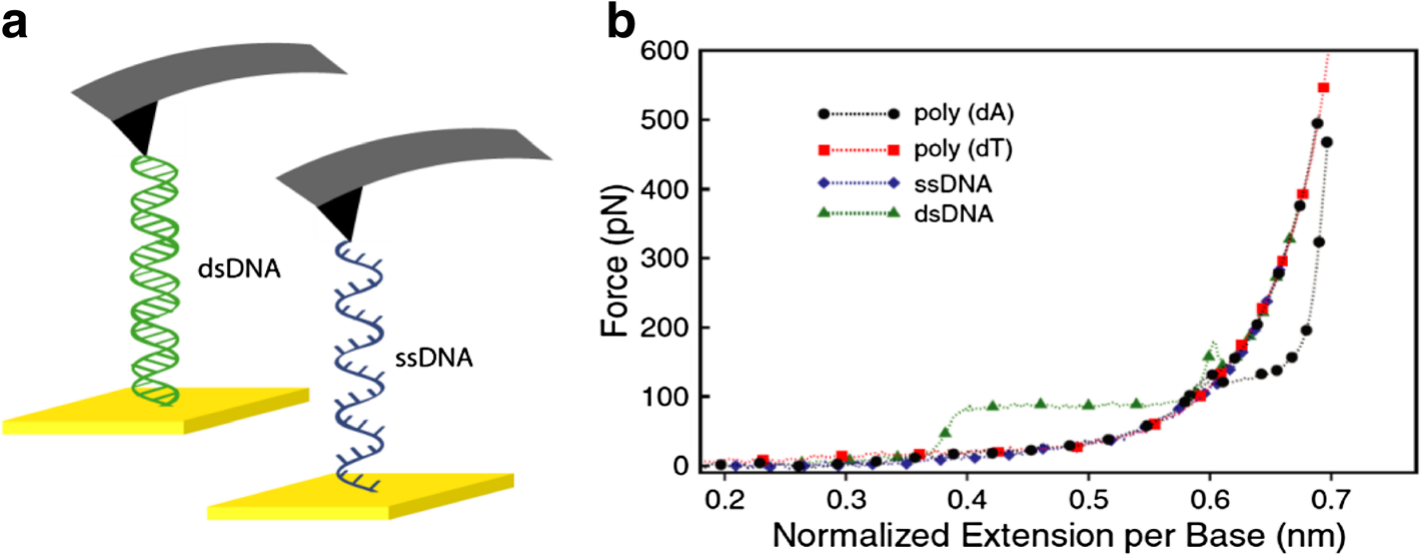
**The stretching of DNA. a**, A schematic of pulling dsDNA and ssDNA by AFM. **b**, Force-extension curves of poly(dA), poly(dT) and λ-phage ssDNA and dsDNA. The curves indicate that transitions occur at 0.6 nm base separation for both dsDNA and poly(dA), but not poly(dT) or λ-phage ssDNA. Adapted from Ref. [41].

AFM is used to pull single-stranded DNA (ssDNA) and dsDNA molecules, and measure the force associated with the conformational changes (Figure 3). DNA melting transitions were characterized by repeatedly stretching and relaxing an individual double-stranded λ-DNA molecule [40]. A force-induced transition between *B* form DNA (*B*-DNA) and *S* form DNA (*S*-DNA), prior to dsDNA melting, was observed [40]. The mechanical properties of the various conformations, *B*-DNA, *S*-DNA, and ssDNA, were quantified using the FJC and WLC polymer elasticity models, and were shown to agree well with expectations from previous experiments and theory.

Poly(dA), a single-stranded DNA composed of uniform A bases [41], has also been studied with force measurements, and was found to have multiple overstretching pathways, with the molecule being able to hop between these two states. These results suggest that poly (dA) has a novel conformation when highly stretched, and the unique conformation makes poly(dA) more stable at large extensions. These unique properties of poly(dA) may play a role in biological processes such as gene expression. Taken together, these results demonstrate that single molecule force measurement allows us to quantify the elastic and thermodynamic properties of biological macromolecules, and the technique may ultimately be developed into a tool for drug screening.

### Cell and tissue mechanics

#### Force-mediated self-patterning of myofibril

**Figure 4:**
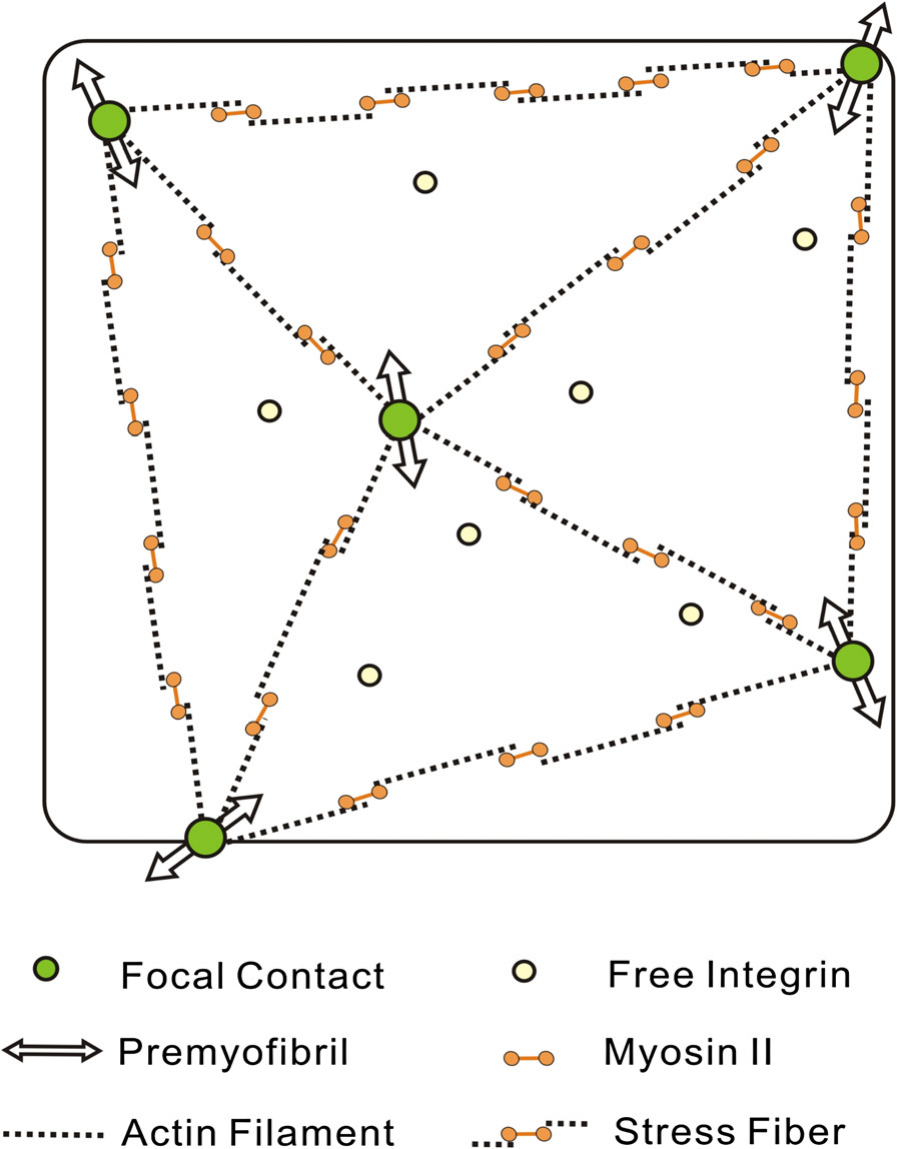
**A schematic for the self-organization of myofibrils.** A single myocyte is patterned into a square shape to bias the distribution of focal contacts (green) at the corners. The contractile units, stress fibers, connect focal contacts to one another. The final pattern of myofibril is predicted to result from the coordination of the geometrical cues and the self-alignment of stress fibers.

**Figure 5:**
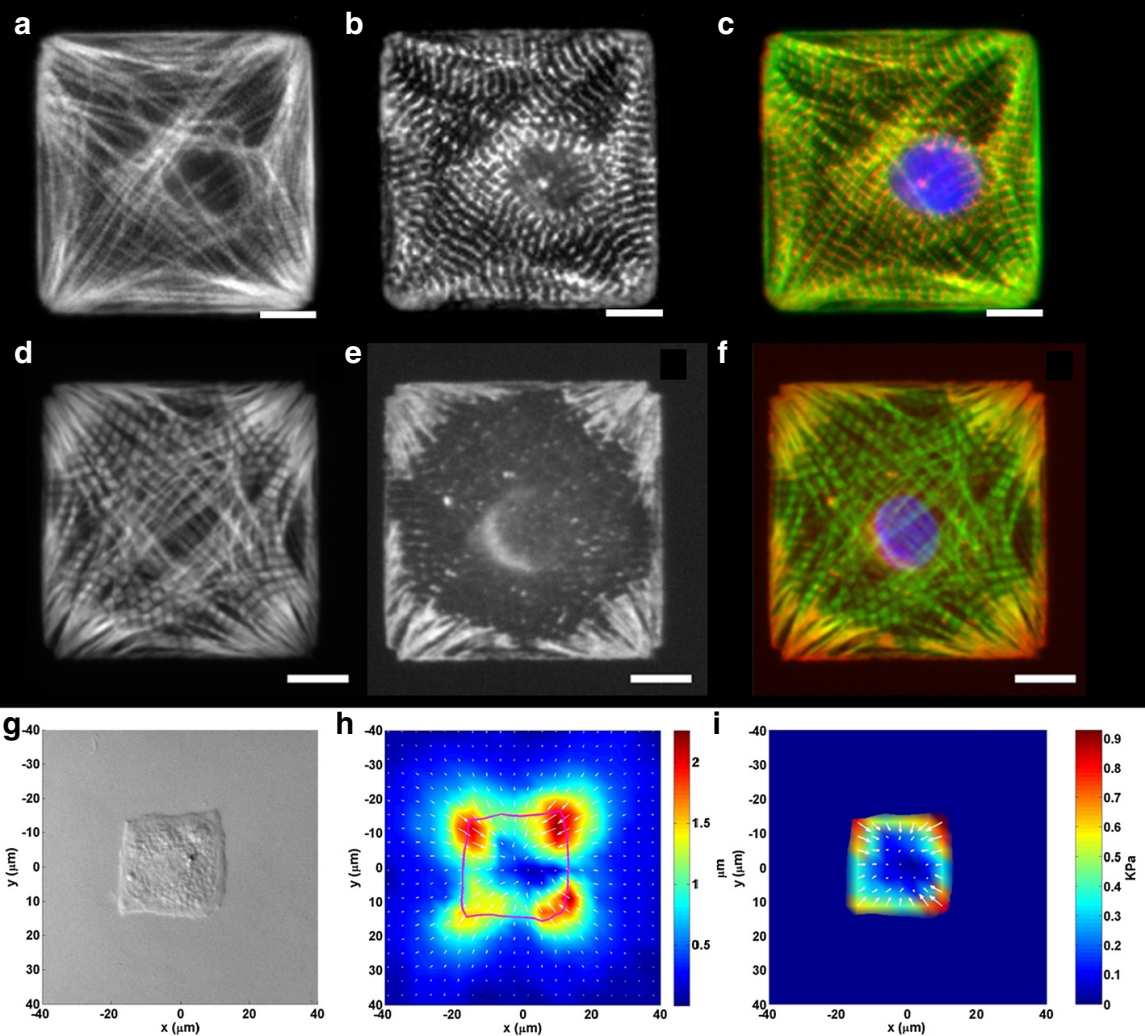
**Immunostains and traction force map of square cardiac myocytes.** Actin (**a**, **d** and green in **c**, **f**) stained in a square myocyte was seen to be aligned along the diagonal, while sarcomeric α-actinin (**b** and red in **c**) marks the z-lines of the sarcomeres. Vinculin stains (**e** and red in **f**) show that this protein aggregates to corners of a square, with fibril-like structures appearing to emanate from the internal angle and radiating towards the center of the cell. The chromatin was stained with blue in **c** and **f**. Scale bar: 10 μm. The contractile traction measurement (**i**) shows that the contraction of myofibrils is centripetal with the highest traction concentrated at the corners, correlating with the distribution of the vinculin (**g**: DIC image; **h**: displacement field; **i**: contractile traction field). Adapted from Ref. [4].

Living cells continuously consume energy to organize and maintain asymmetric architectures dictating their functions. One specific example is the orientation of myofibrils, which is a cord-like structure consisting of several different types of filamentous proteins that are organized into a regularly repeated subunit, the sarcomere. Myofibril matures in a force-dependent manner [4]. Within cells, force is maximized when all the contractile units are aligned. Cells develop contractile units such as stress fibers by establishing cell-substrate contacts called focal adhesions. It has been shown that the geometry of cell periphery determines the distribution of focal adhesions [42,43]. Thus, it is likely that the patterning of myofibrils is due to the coordination of extracellular geometrical cues and the self-alignment of intracellular contractile units (Figure 4). This was supported by computation simulation and micro-patterning experiments [4] (Figure 5).

#### Mechanical processes pattern cell shape and tissue architecture

In most tissues, cells can iteratively pattern morphogenetic units of similar size into complex forms. This occurs at both single- and multi-cell levels. For example, in the morphogenesis of a growth cone, single neuron cells form multiple, regularly separated neurites, which then differentiate into axon and dendrites [44]. Likewise, repetition of multi-cellular units is observed in the branching morphogenesis of tubular organs including lungs [45,46], blood vessels [46,47], salivary glands [48], mammary glands [46], and renal ducts [46,49,50]. In branching morphogenesis, cells reiteratively branch out from pre-existing cell sheets into the surrounding extracellular matrix (ECM) [46,47]. Similar processes occur in embryo gastrulation, where a group of cells from the ectoderm bend inward to form the endoderm [51].

**Figure 6:**
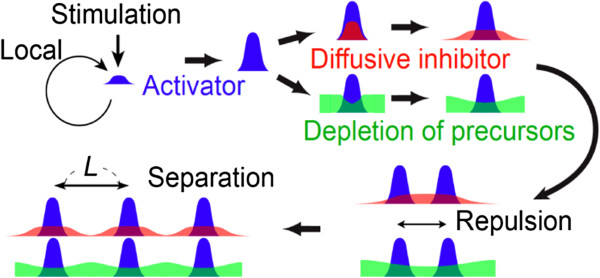
**The schematics of lateral inhibition.** Lateral inhibition requires a short-range activation that self amplifies and a long-range inhibition that inhibits remote activations. The strength of the stimulation and the spatial characteristics of the activation and the inhibition determine the spacing *L*.

One appealing mechanism to control repetitive cell/tissue patterning is the chemical-based reaction–diffusion scheme proposed by Alan Turing (lateral inhibition) [52]. Lateral inhibition relies on the interplay of short-range activation and long-range inhibition (Figure 6). Such inhibition can result from the consumption of precursors or the creation of inhibitors. Lateral inhibition requires that the activation self amplifies, while creating inhibition to suppress other activations. As a result, individual activations mutually repel each other. This leads to a regular spacing, *L*, between neighboring activations. In turn, position cues can spontaneously emerge to pattern cell/tissue into regular/periodic shape.

To form lateral inhibition, it is required that the activation and the inhibition possess different spatial scales. In chemical-based lateral inhibition, this requirement can be achieved by having different diffusion coefficients for molecules that mediate the activation or the inhibition [45,46,50,53-59]. On the other hand, using mechanical processes to generate patterning cues has shown promise as an alternative method.

**Figure 7:**
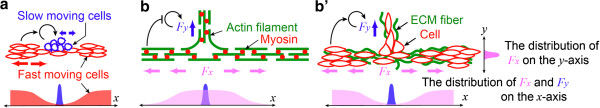
**Models for mechanical force-mediated patterning processes. a**, Different motilities between two types of cells can lead to lateral inhibition. **b**-**b**’, The spatial distributions of an orthogonal pair of mechanical forces, *F*_*x*_ and *F*_*y*_, created by (**b**) myosin or (**b**’) cell motions, are anisotropic along their principle axes, *x* and *y*, respectively.

**Figure 8:**
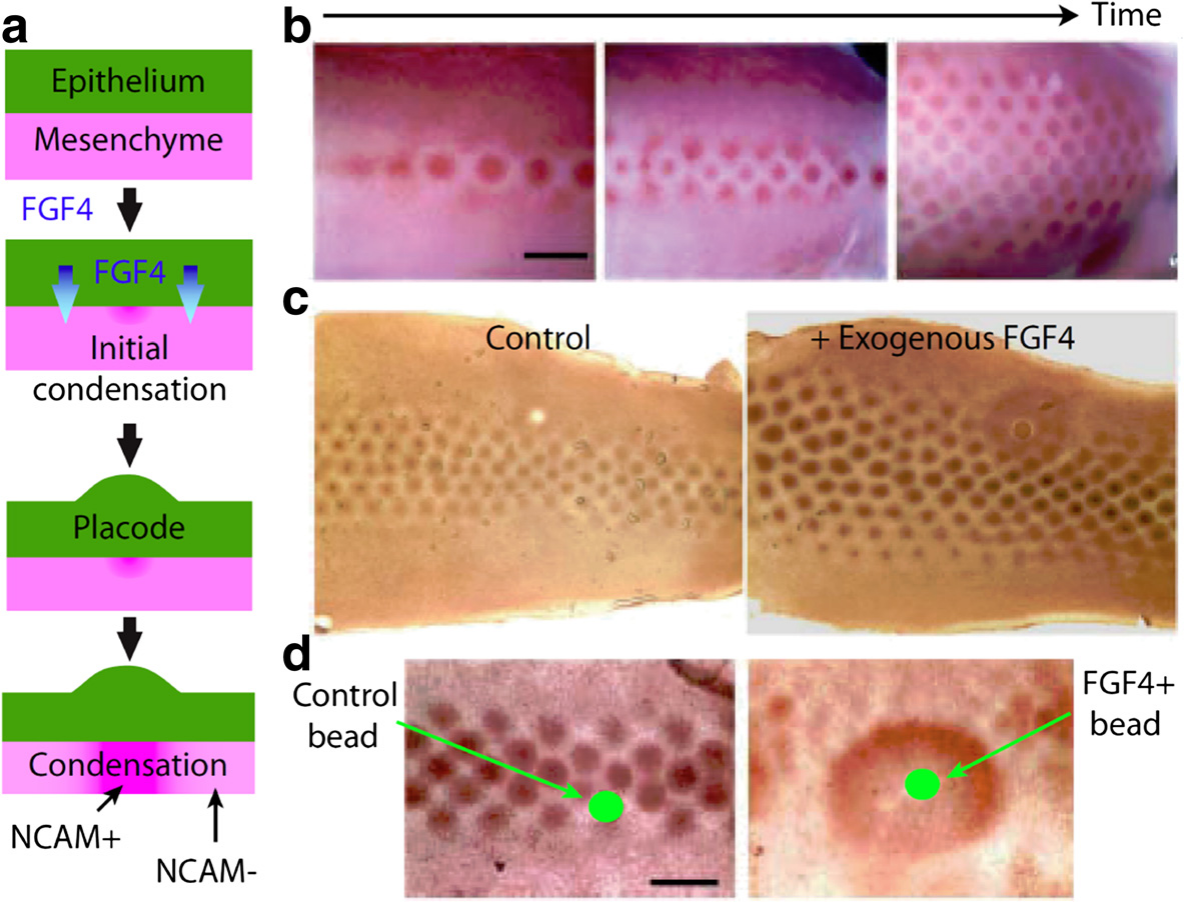
**Epithelium-mesenchyme interaction in skin appendage patterning. a**, The schematics of the epithelial placode formation and the mesenchymal condensation. **b**, The progressive formation of feather buds at chicken embryo. **c**, A homogeneous treatment of FGF4 enlarges feather primodia. **d**, A localized treatment of FGF4 leads to a local enlargement of feather primodia. Scale bar in **b**, **d**: 5 mm. b-d are adapted from Ref. [61].

Compared with chemical-based processes, several features of mechanical processes make them easier to create patterning cues. One example is that cells use differential motility to pattern tissues. For two types of cells that interact with each other (Figure 7a), they exhibit different motility by tuning cytoskeletal mechanics or the expression level of surface receptors that mediate cell-cell or cell-ECM adhesions. Assuming that the slow-moving cells act as the activator and the fast-moving cells act as the inhibitor, we can then see how their interplay leads to lateral inhibition. This can be found in the patterning of skin appendages, such as hair follicles [54] and feather buds [60], where mesenchymal cells interact with epithelium and change motility by expressing different amounts of receptor N-CAM (Figure 8).

**Figure 9:**
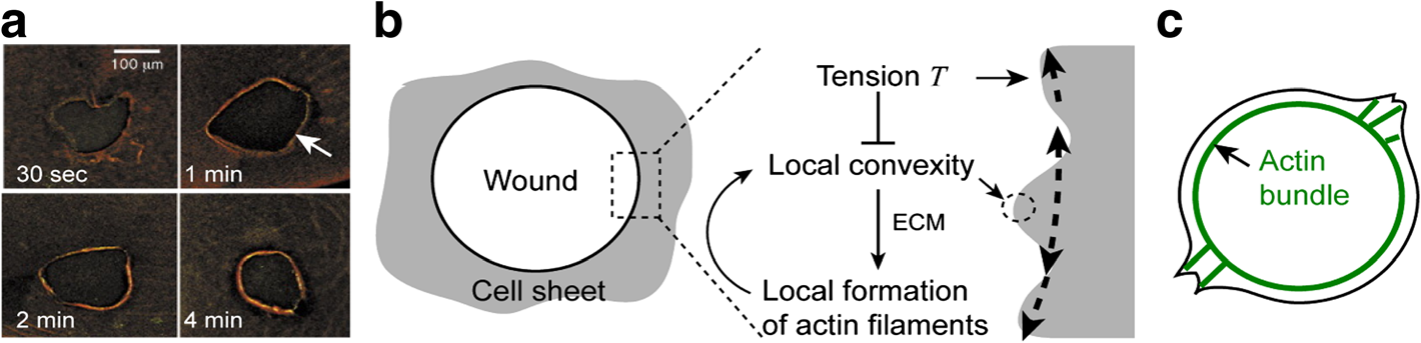
**Models for force-mediated patterning process in wound healing and cell shape regulation. a**, The time course of wound healing in xenopus oocyte. Adapted from Ref. [63]. White arrows indicate actomyosin bundles (purse-string). **b**-**c**, Models for how (**b**) cell sheet and (**c**) individual cells use the interplay of tension along actomyosin bundles and the geometry-dependent positive feedback of cell protrusions to form patterning cues.

Another feature that makes mechanical force useful in patterning processes is its vectorial nature, *i.e*., spatial anisotropy (Figure 7b, b’). This is different from molecular diffusion and can easily create patterning cues in high-dimensional space. For a mechanical process that involves two orthogonal forces, both forces propagate along linear polymers such as actin filaments or ECM fibers (Figure 7b, b’). These forces can be generated by either motor protein myosin II within individual cells or by moving cells at the ECM. In contrast to isotropic molecular diffusion, the magnitude of each force lasts for a long range along its own principle axis, but limited in the others. This effect leads to a difference of spatial scales for the distribution/dispersion of forces between orthogonal axes. When the mechanical force is coupled with chemical signaling such as the process of mechanotransduction [62], the traction force created by one cell can act on other cells and allow them to produce more traction forces along the same axis, which results in an amplification loop. The amplification loop and the difference in spatial scales (for the dispersion of forces) then provide a foundation for lateral inhibition to occur along one of the principle axes. Typical examples include the closure of wound and the patterning of cell shape. In embryos, the healing of wounds is primarily mediated by the contraction of actomyosin bundles within multiple cells along the wound edge, *i.e.*, a purse-string mechanism [63] (Figure 9a), while individual cells at the wound edge stochastically form lamellipodia in the direction of the wound [64]. The formation of lamellipodia occurs in a geometry-dependent manner [42,43] within individual cells, while the contraction of purse-string occurs across multiple cells along the wound edge, leading to a difference in spatial scales between these two processes. In addition, the tension of actomyosin bundle provides a bending modulus to suppress cell protrusion [65]. As a result, it is expected that the long-range transmission of tension along the wound edge and the geometry-dependent amplification of lamellipodia in the direction of wound form a foundation to create patterning cues in wound healing (Figure 9b). A similar idea can be applied to the patterning of cell shape (Figure 9c). A mathematical model based on assumptions analog to the proposed concept is discussed in Ref. [65].

**Figure 10:**
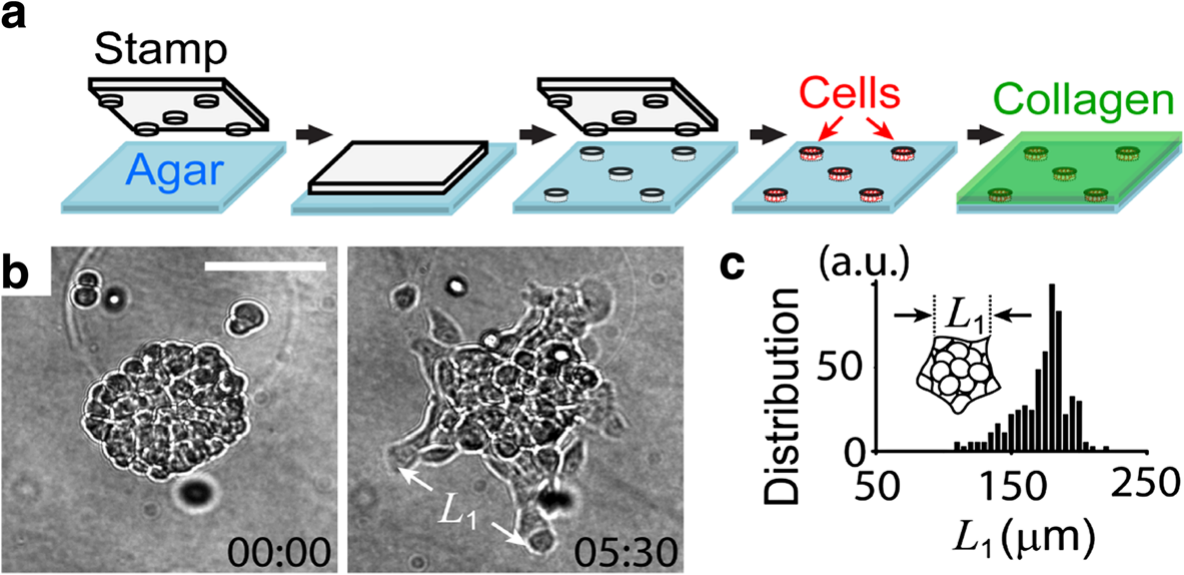
**Regular spacing in the invasion of tumor colonies. a**, Experimental setup. Tumor cells (the Human MCF-7) were seeded in micro-patterned traps followed by the overlay of type I collagen gel (1 mg/ml) to induce cell migration. **b**, Representative invasion patterns. The pattern became random if cells were treated with myosin inhibitor Blebbistatin (10 μM). Time is in hour and minute. Scale bar: 200 μm. **c**, Distribution of inter-branch distance *L*_1_ after 6 h. N = 100 and data are normalized to the maximum.

**Figure 11:**
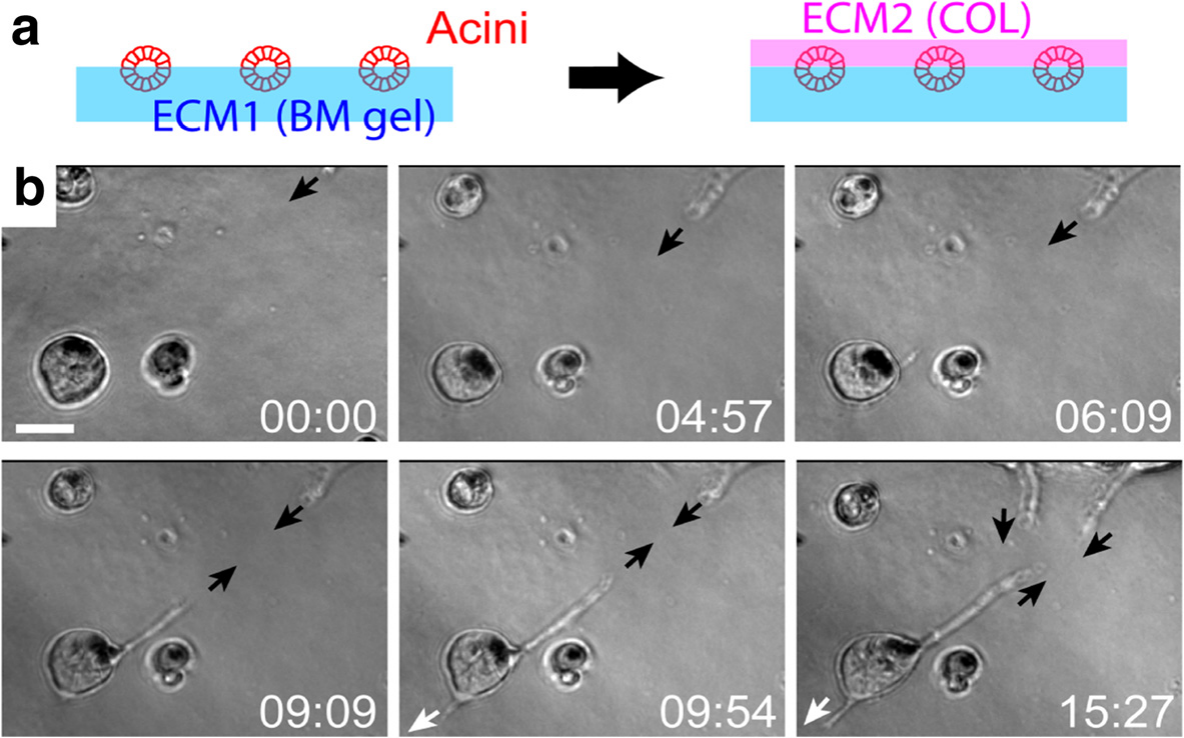
**Force as an attractive morphogen. a**, Experimental setup. Epithelial cells (The Human MCF-10A) were spread on basement membrane (BM) gels (ECM1) to form epithelial acini, followed by the overlay of collagen (ECM2) on top. **b**, Representative branch induction by mechanical attraction (black arrow) between acini. Note branches originated from different acini attract each other, while branches originated from the same acinus move in opposite direction (black and white arrows). The attraction was inhibited when cells were treated with myosin inhibitor Blebbistatin (10 μM). Time is in hour and minute. Scale bar: 100 μm.

Perhaps the most important feature of mechanical forces is that they can continuously propagate between and across cells through cytoskeletons, intercellular adhesions [66,67], and ECM [68]. This provides long-range communication across the multi-cellular system. Likewise, patterning cues mediated by mechanical force can propagate from single-cell to multiple-cell levels without the transformation of the patterning information by biochemical cues, such as morphogens. Figure 10 shows an example where multi-cellular protrusions (formed by actin filament polymerization [69]) are separated from each other to form regular spacing, as one would expect in lateral inhibition. Figure 11 shows an example where epithelial acini use mechanical force as an “attractive morphogen” to induce branch formation.

**Figure 12:**
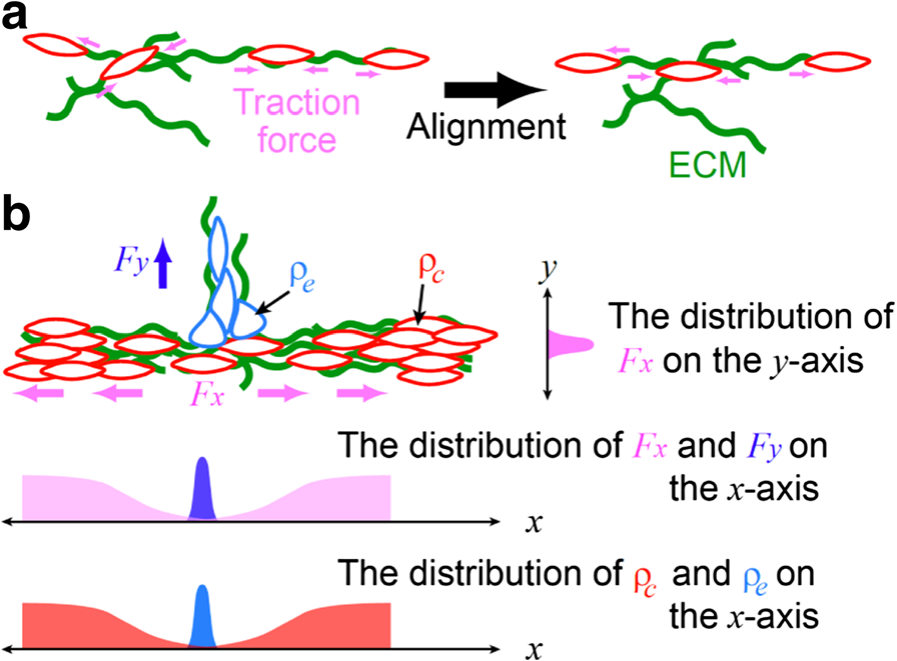
**Model for force-mediated branch patterning. a**, The long-range traction forces (pink arrows) align cell motions in the same orientation. **b**, The spatial distributions of traction forces, *F*_*x*_ and *F*_*y*_, created by the constrained (red) and escaping (blue) cells, respectively, are anisotropic along *x* and *y* axes. Here, the constrained cells move along aligned linear ECM polymers (green) such as type I collagen (COL) fibers. On the other hand, the escaping cells are attracted by un-aligned ECM polymers. The densities of the constrained and escaping cells are *ρ*_*c*_ and *ρ*_*e*_, respectively.

The propagation of forces over multiple cells further reinforces the long-range effect for lateral inhibition. This will be very useful for creating large-scale coordination in tissue development and homeostasis. Figure 12 illustrates an example for how mechanical interactions between cells and ECM can help the creation of patterning processes. Imagine a group of cells surrounded by linear ECM polymers such as type I collagen (COL) fibers. Through spontaneous migration, these cells create traction forces between each other via the intercellular adhesions and the ECM [68] (Figure 12a). The propagation of forces provides a long-range control across multiple cells to align their locomotion in the same orientation. In addition, it helps the alignment of collagen fibers. As a result, cells are constrained to move along aligned collagen fibers (Figure 12b). Now, imagine that a fraction of cells escapes through interaction with non-aligned collagen fibers. Traction force created by escaping cells can then act at the constrained cells to change their direction of motions through mechanotransduction [62]. This effect allows the constrained cells to escape from the aligned collagen fibers, and becomes more significant as more constrained cells become the escaping cells, leading to a local amplification of escape. Taken together, the long-range alignment and the local amplification of escape form a foundation to create patterning cues.

## Conclusion

The effect of mechanical force on biological materials differs from that of chemical force in that it depends both on the force-molecular interactions and the structure of underlying substrate. This opens a door for using nanotechnology to control the molecular, cellular, and tissue structure, function, and assembly by changing the topology and structure of the environment. There are several advantages of using physical versus chemical forces to control the response of biological materials and complexes. For example, mechanical force is nonspecific, which does not depend on the types of molecules, cells, and tissues involved, so the effect and design principle is universal. Furthermore, unlike chemical signaling, the non-specificity of mechanical forces allows them to be directly combined, and the effect may be amplified by increasing the magnitude of force applied. These features, along with the relatively simple processes required for generating mechanical processes, make mechanical force a promising tool to control and manipulate biological materials.

## Abbreviations

(ECM): Extracellular matrix; (FJC): Freely-jointed chain; (eFJC): Extensible FJC; (WLC): Wormlike chain; (ssDNA): Single-stranded DNA; (dsDNA): Double-stranded DNA; (B-DNA): *B* form DNA; (S-DNA): *S* form DNA; (Ig): Immunoglobulin; (FN3): Fibronectin type 3; (BM): Basement membrane; (COL): Collagen.

## Competing interests

The authors declare that they have no competing interests.

## Authors’ contributions

CG conceived the study, participated in its design, and drafted the manuscript. NCH carried out the experiments and drafted the manuscript. SSW and EWF drafted the manuscript. CHK conceived the study, participated in its design, and drafted the manuscript. All authors read and approved the final manuscript.
